# Permanent pacing in a very long‐term follow‐up after orthotopic heart transplantation: A matter of when or why?

**DOI:** 10.1111/anec.12979

**Published:** 2022-06-07

**Authors:** Emyal Alyaydin, Christian Pogoda, Angelo Dell'Aquila, Gerrit Frommeyer, Juergen R. Sindermann, Holger Reinecke, Izabela Tuleta

**Affiliations:** ^1^ Department of Cardiology I ‐ Coronary and Peripheral Vascular Disease, Heart Failure University Hospital Muenster Muenster Germany; ^2^ Department of Cardiothoracic Surgery University Hospital Muenster Muenster Germany; ^3^ Department of Cardiology II – Electrophysiology University Hospital Muenster Muenster Germany

**Keywords:** conduction disturbances, orthotopic heart transplantation, pacemaker dependence, permanent pacemaker

## Abstract

**Background:**

Orthotopic heart transplantation (OHT) is associated with a high incidence of conduction disturbances (CD) leading to permanent pacemaker (PPM) implantation. However, the improved posttransplant survival raises the question about the pacemaker dependence (PD) in a prolonged follow‐up.

**Hypothesis:**

The prevalence of PPM in OHT is high but not all patients are PD in a very long‐term follow‐up. Device implantation has no prognostic relevance.

**Methods:**

We performed a retrospective analysis of patient medical records focusing on device interrogation data at the most recent follow‐up.

**Results:**

The study population consisted of 183 patients with a mean follow‐up of 15.0 ± 6.8 years. One‐fourth of the patients had undergone PPM implantation (*n* = 49, 26.8%). Among these, two‐thirds were PD at last follow‐up (*n* = 32, 65.3%). PPM was more often in biatrial OHT and cardiac allograft vasculopathy (OR 3.0, 95% CI 1.26–7.29, *p* = .013 and OR 2.0, 95% CI 1.03–3.87, *p* = .041, respectively). Early sinus node dysfunction (SND) was the most persistent CD. PPM was associated with a poorer outcome in OHT (HR 1.9, 95% CI 1.06–3.46, *p* = .031) and a higher rate of fatal septicemia (HR 5.1, 95% CI 1.41–18.14, *p* = .013).

**Conclusions:**

One‐fourth of the OHT recipients develop CD requiring PPM implantation, although one‐third among these are not PD in follow‐up. Early SND is associated with a higher rate of PD. PPM is associated with an inferior prognosis.

## Graphical abstract

Orthotopic heart transplantation is associated with a high incidence of conduction disturbances requiring permanent pacemaker (PPM) implantation. However, a significant amount of these resolve in a very long‐term follow‐up. Early sinus node dysfunction is the indication associated with the highest rate of pacemaker dependence. Patients undergoing PPM implantation have an inferior prognosis.

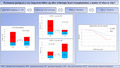

## 1 INTRODUCTION

Orthotopic heart transplantation (OHT) is associated with a high rate of conduction disturbances (CD). Among CD requiring permanent pacemaker (PPM) implantation, sinus node dysfunction (SND) is more frequent in the immediate posttransplant period, whereas atrioventricular conduction disturbances (AVCD) are more common in long‐term follow‐up. Previous studies have reported an incidence of PPM implantation in OHT of 2%–24%.(Mallidi & Bates, 2017; Rivinius et al., 2019; Woo et al., 2008) Nevertheless, rhythm disturbances occurring after OHT might be due to surgical trauma and may potentially subside over time, but data regarding the persistence of PPM in OHT and its influence on patient outcomes remain scarce. However, statistics have indicated that Germany is among the European countries with the highest rates of cardiac device implantations.(Raatikainen et al., 2015) When assessing PPM indication in OHT recipients, one should take into consideration that these patients have multiple comorbidities, often undergo invasive procedures, and are under lifelong immunosuppression, thus increasing their risk of potential device‐related infections.(Paterson et al., 1998; Sherman‐Weber et al., 2004)

Therefore, we sought to elucidate the extent to which patients who had undergone PPM implantation were pacemaker dependent (PD) in a very long‐term follow‐up after OHT. Additionally, we examined the prognostic relevance of PPM and PD.

## 2 METHODS

We performed a retrospective analysis of patient data collected at the most recent follow‐up in an outpatient setting. The on‐site examination of OHT recipients was planned in 3‐month intervals and consisted of a brief history, clinical examination, electrocardiogram, and laboratory testing at every presentation, whereas transthoracic echocardiogram and pacemaker interrogation were performed every 6 months.

**FIGURE 1:**
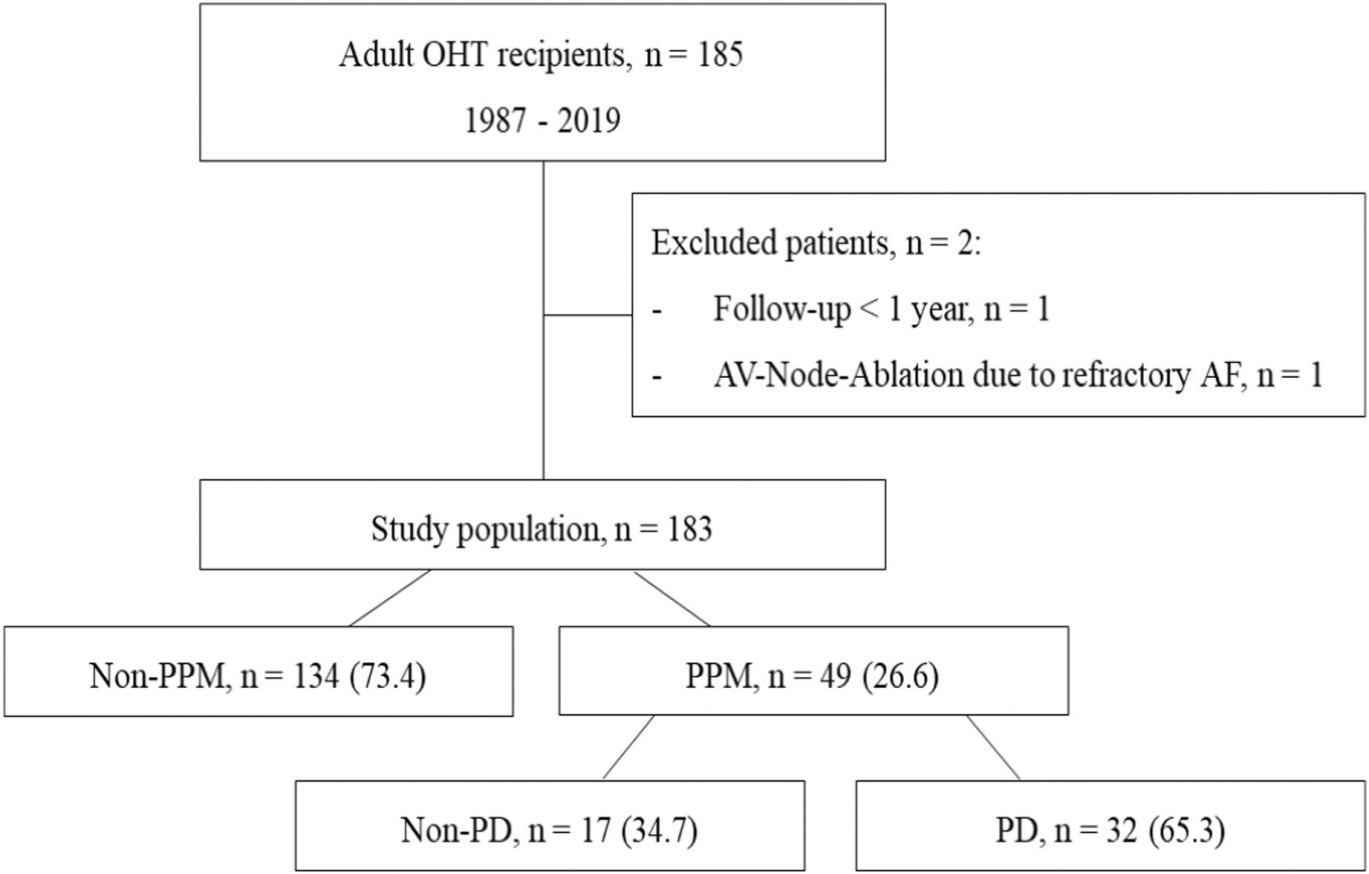
Flowchart of the study. Data are presented as number (percentage). Abbreviations: AV‐Node‐Ablation ‐ atrioventriculat node ablation; OHT, orthotopic heart transplantation; PPM, permanent pacemaker; PD, pacemaker dependence

Among the 185 OHT recipients, one patient was excluded from the analysis because of a follow‐up <1 year. Additionally, one patient was not included, as the pacemaker dependence was secondary to AV node ablation in refractory atrial fibrillation (Figure 1). We identified 49 patients who had undergone PPM implantation. According to the results of the last device interrogation, patients with PPM were stratified into two groups: PD and non‐PD. No need for pacing was defined as atrial and/or ventricular pacing percentages <0.1% at the last assessment. Furthermore, we differentiated between early‐PPM (<1 year after OHT) and late‐PPM implantation (≥1 year after OHT). The underlying rhythm disturbances were further subdivided into two groups – SND and AVCD (Figure 1).

Patients in whom PPM explanation was performed because of no need for pacing or device‐related complications were included in the non‐PD group (*n* = 5). Among these, device‐related endocarditis was suspected in one subject and Twiddler's syndrome was observed in a further case.

Additionally, in one case, a device explantation because of an infection with consequent reimplantation due to PD was performed.

The study was performed in compliance with the Declaration of Helsinki, and data sampling was approved by the local ethics committee (2019‐021‐f‐S).

### 2.1 Statistics

Statistical analysis was conducted in IBM SPSS Statistics software, version 27. Continuous variables were expressed as mean ± standard deviation (SD) and were further assessed with Student's t‐test. Categorical variables were given as numbers and percentages and were tested by chi‐square test. The evaluation of potential risk factors for pacemaker dependence was performed with univariable and multivariable cox‐regression analyses. For all statistical analyses, *p* < .05 was defined as significant.

## 3 RESULTS

### 3.1 Demographics

**FIGURE 2:**
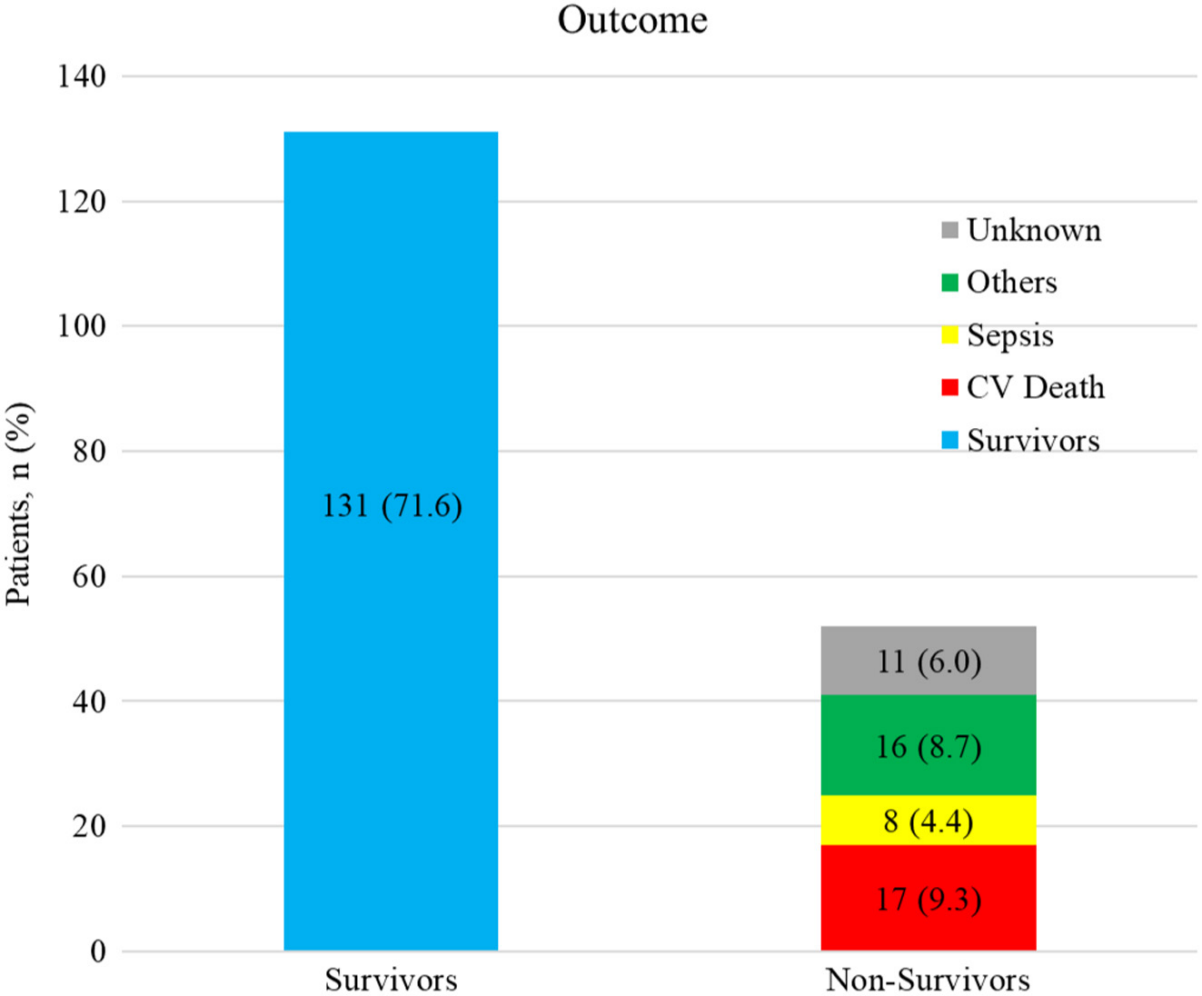
Outcome. Abbreviation: CV death, Cardiovascular death

The study population consisted of 183 patients with a mean follow‐up of 15.0 ± 6.8 years and a mean age at the time of OHT of 44.7 ± 15.5 years. Approximately, one‐fifth of the overall population were women (*n* = 37, 20.2%). According to the survival status, almost three‐quarters of the recipients were alive at the time point of the study (*n* = 131, 71.6%), (Figure 2). Common etiologic contributors in the pretransplant stage were ischemic and dilated cardiomyopathy. More than half of the patients had rejection episodes in the time course after OHT. Almost 40% of the population had rejections requiring therapy (Grade ≥ 2R), according to the revised classification of the International Society for Heart and Lung Transplantation (ISHLT) from 2004.(Stewart et al., 2005) We observed no statistically significant differences regarding the aforementioned determinants between the groups with/without PPM and PD/non‐PD. Patients with PPM more often had cardiac allograft vasculopathy (CAV), defined as ≥ ISHLT CAV_1_ (Table 1a,b),.(Mehra et al., 2010) Most of the patients had a dual‐chamber pacemaker (*n* = 35, 71.4%); single‐chamber devices were predominantly with a ventricular lead (*n* = 10, 20.4%) and rarely with an atrial lead (*n* = 4, 8.2%). Additionally, we observed no gender‐related differences in the frequency of PPM (*n* = 40, 27.4% in male vs. *n* = 9, 24.3% in female), although females were underrepresented in our patient population.

**TABLE 1:** Patient characteristics

(a) Overall patient population			
Main patient characteristics	Non‐PPM *n* = 134 (73.2)	PPM *n* = 49 (26.8)	*p* value
1. Demographics			
Age at HTx, years	43.8 ± 15.8	47.2 ± 14.5	.190
Ischemic time, min^a^	193.4 ± 51.7	179.6 ± 53.9	.136
Follow‐up, years	15.3 ± 7.3	14.3 ± 5.1	.289
Donor age, years^b^	30.79 ± 12.4	31.1 ± 15.1	.906
Male, *n* (%)	106 (79.1)	40 (81.6)	.836
Survivors, *n* (%)	104 (77.6)	31 (63.3)	.059
2. Pretransplant heart disease			
Ischemic cardiomyopathy, *n* (%)	45 (33.6)	21 (42.9)	.297
Dilated cardiomyopathy, *n* (%)	62 (46.3)	21 (42.9)	.739
Others, *n* (%)	27 (20.1)	7 (14.3)	.520
3. Transplant technique			
Biatrial, *n* (%)	89 (66.4)	42 (85.7)	.010*
Bicaval, *n* (%)	37 (27.6)	6 (12.2)	.031*
Total, *n* (%)	8 (6.0)	1 (2.0)	.448
3. Rejections and CAV			
Rejection episodes, *n* (%)	73 (54.5)	29 (59.2)	.617
Rejections requiring therapy, *n* (%)	46 (34.3)	22 (44.9)	.227
CAV, *n* (%)	51 (38.1)	27 (55.1)	.044*
4. Clinical and laboratory examination			
NYHA class >1, *n* (%)	101 (75.4)	38 (77.6)	.847
NT‐proBNP, pg/ml	4094.2 ± 7668.4	6107.2 ± 8396.4	.127
5. Echocardiographic assessment			
LVEF, (%)	57.1 ± 6.8	56.8 ± 9.4	.835
TAPSE, mm	16.4 ± 3.6	16.1 ± 5.7	.735

(b) PPM cohort			
Main patient characteristics	Non‐PD *n* = 17 (34.7)	PD *n* = 32 (65.3)	*p* value
1. Demographics			
Age at HTx, years	44.2 ± 17.7	48.7 ± 12.5	.307
Ischemic time, min^a^	167.4 ± 64.6	186.2 ± 46.9	.268
Follow‐up, years	16.0 ± 4.8	13.4 ± 5.1	.088
Donor age, years^b^	27.7 ± 13.3	32.8 ± 15.8	.284
Male, *n* (%)	13 (76.5)	27 (84.4)	.700
Survivors, *n* (%)	11 (64.7)	20 (62.5)	1.000
2. Pretransplant heart disease			
Ischemic cardiomyopathy, *n* (%)	8 (47.1)	13 (40.6)	.765
Dilated cardiomyopathy, *n* (%)	5 (29.4)	16 (50.0)	.229
Others, *n* (%)	4 (23.5)	3 (9.4)	.217
3. Transplant technique			
Biatrial, *n* (%)	15 (88.2)	27 (84.4)	1.000
Bicaval, *n* (%)	1 (5.9)	5 (15.6)	.650
Total, *n* (%)	1 (5.9)	0 (0.0)	.347
3. Rejections and CAV			
Rejection episodes, *n* (%)	9 (52.9)	20 (62.5)	.555
Rejections requiring therapy, *n* (%)	6 (35.3)	6 (18.8)	.296
CAV, *n* (%)	12 (70.6)	15 (46.9)	.140
4. Clinical and laboratory examination			
NYHA class >1, *n* (%)	11 (64.7)	27 (84.4)	.156
NT‐proBNP, pg/ml	6623.1 ± 8334.7	5833.1 ± 8548.8	.758
5. Echocardiographic assessment			
LVEF, (%)	57.5 ± 9.6	56.3 ± 9.4	.673
TAPSE, mm	15.9 ± 4.1	16.1 ± 6.5	.916

*Note*: Data are presented as mean ± standard deviation or number (percentage).

Abbreviations: CAV, cardiac allograft vasculopathy; HTx, heart transplantation; LVEF, left ventricular ejection fraction; NT‐proBNP, N‐terminal prohormone of brain natriuretic peptide; NYHA, New York Heart Association Classification; PPM, permanent pacemaker; TAPSE, Tricuspid annular plane systolic excursion.

^a^ Data available in *n* = 155 (85%) of the population.

^b^ Data available in *n* = 151 (82.5%) of the population.

^*^ *p* < .05 is defined as statistically significant.

### 3.2 Transplantation technique and PPM

Biatrial transplant technique was associated with a higher rate of PPM implantation in a univariate logistic regression analysis (OR 3.0, 95% CI 1.26–7.29, *p* = .013), whereas in bicaval OHT, the need for PPM was significantly lower (OR 0.4, 95% CI 0.14–0.93, *p* = .035). A total approach was preferred only in a limited number of patients. However, we observed no relevant influence of the surgical blueprint on PD at the last follow‐up (OR 0.7, 95% CI 0.12–4.17, *p* = .714 for biatrial and OR 3.0, 95% CI 0.32–27.67, *p* = .341 for bicaval transplant technique).

### 3.3 Timing‐related indication for PPM

**FIGURE 3:**
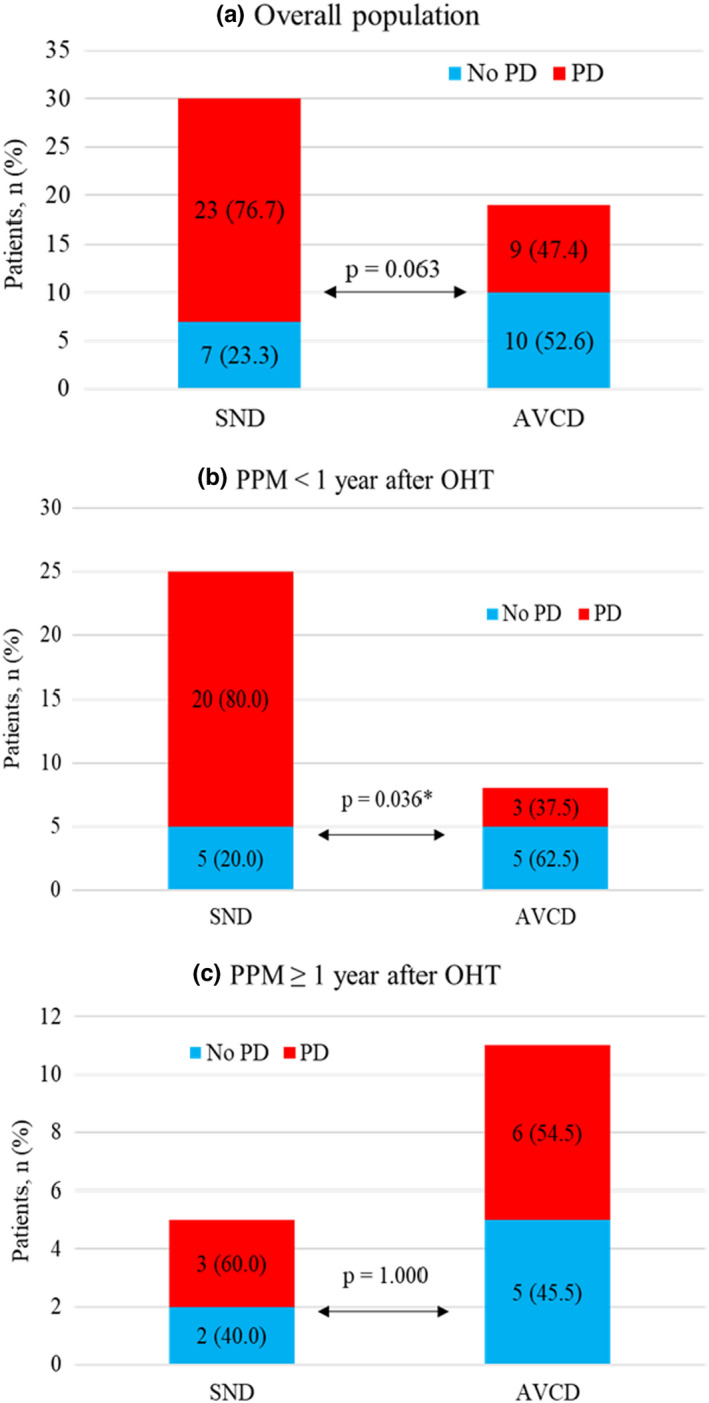
Conduction disturbances and pacemaker dependence (PD). (a) Overall population. (b) PPM <1 year after OHT. (c) PPM ≥1 year after OHT. Data are presented as number (percentage). OHT – Orthotopic heart transplantation, Abbreviations: AVCD, atrioventricular conduction disturbances; PD, Pacemaker dependence; PPM, Permanent pacemaker; SND, Sinus node dysfunction

We detected no indication‐related influence on PD in the overall population. Nonetheless, after stratification according to the time point of PPM implantation, AVCD early after OHT were found to be more likely to resolve. In contrast, early SNDs were still associated with PD in more than 80% of the patients. These differences were not observed in late‐PPM (Figure 3).

### 3.4 Cardiac parameters and PPM

Regardless of PPM implantation or PD, there were no statistically significant intergroup disparities concerning the biventricular allograft function expressed as left ventricular ejection fraction (LVEF) and tricuspid annular plane systolic excursion (TAPSE). Additionally, we observed no correlation between the ventricular pacing percentage and the LVEF (*p* = .357). The N‐terminal pro b‐type natriuretic peptide (NT‐proBNP) levels were also comparable between groups. Furthermore, an assessment of the device‐related consequences on the functional status of the recipients according to the New York Heart Association (NYHA) classification revealed no relevant differences between the groups (Table 1).(The Criteria Committee of the New York Heart Association, 1994)

### 3.5 CD in the non‐PPM population

Regardless of PPM status, the rate of conduction disturbances was higher in the OHT recipients than in the general population. The most common CD in non‐PPM patients was a right bundle branch block (RBBB) (*n* = 74, 55.2%), followed by a left anterior fascicular block (LAFB) (*n* = 23, 17.2%), and a first‐degree atrioventricular block (AV block) (*n* = 7, 5.2%). A left bundle branch block was less frequent (*n* = 2, 1.5%).

### 3.6 Long‐term follow‐up

**FIGURE 4:**
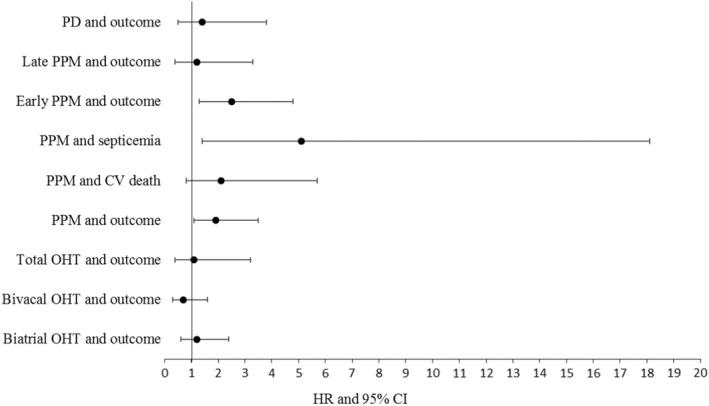
Outcome based on transplant technique, PPM and PD in OHT in a univariate cox‐regression analysis. Abbreviations: OHT, Orthotopic heart transplantation; PD, Pacemaker dependence; PPM, Permanent pacemaker

Although we do not have data on the incidence of cardiac syncope following OHT, the mortality rate was higher in patients with PPM (15‐year mortality rate in PPM 14.5% vs. 11.9% in the non‐PPM population, *p* = .026). Furthermore, PPM requirement was found to be a significant factor influencing mortality in a long‐term follow‐up after OHT in a univariate (HR 1.9, 95% CI 1.1–3.5, *p* = .031) as well as in a multivariate logistic regression analysis after adjustment for CAV, transplant technique, and left ventricular ejection fraction (HR 2.0, 95% CI 1.1–3.7, *p* = .033). Nevertheless, we observed no relevant impact on cardiovascular death in the same setting (HR 2.1, 95% CI 0.8–5.7, *p* = .126), but on mortality due to systemic infections and septicemia (HR 5.1, 95% CI 1.4–18.1, *p* = .013). Furthermore, after stratification according to the time point of PPM implantation, early‐PPM was associated with poorer outcomes (HR 2.5, 95% CI 1.3–4.8, *p* = .004), whereas late‐PPM did not influence mortality (HR 1.2, 95% CI 0.4–3.3, *p* = .737; Figure 4).

## 4 DISCUSSION

To our knowledge, this is the first study evaluating the potential prognostic effects of PPM in a very long‐term follow‐up after OHT. An additional advantage is the use of device interrogation data to assess the need for permanent pacing.

### 4.1 A history lesson

As previously reported, biatrial OHT was associated with a higher rate of CD requiring PPM implantation, whereas in bicaval OHT, the incidence of CD was significantly lower.(Jones et al., 2011; Schnoor et al., 2007) No reliable statement can be made about the total transplant technique because our experience was limited to a small number of cases. On the one hand, this approach creates no anatomical substrate for CD, but on the other hand, it is often associated with a prolonged ischemic time with potential consequences for the graft function.(Morgan & Edwards, 2005) Although we did not observe relevant differences in donor age between groups, there was a larger gap in PD/non‐PD compared to PPM/non‐PPM, thus indicating the potential of advanced donor age to cause persistent CD. In addition, CAV was more prevalent in the PPM group indicating the potential of the ischemic injury to cause CD, but we observed relation to the type of CD in contrast to previous studies (*p* = .155).(Cui et al., 2003)

### 4.2 Timing and indication

In line with prior reports, approximately 25% of the patients required PPM, and the indication distribution was dominated by early SND and late AVCD.(Jones et al., 2011; Woo et al., 2008) Previous research on the role of the type and timing of CD in PPM have indicated that early CD are more likely to resolve and to not require PPM, whereas late CD were considered more persistent.(Rivinius et al., 2019) The indication paradox in our study may be due to the most frequent use of the biatrial approach and the higher incidence of associated CD. Additionally, 90% of the SNDs were diagnosed in patients with biatrial OHT, the transplant technique with the greatest potential to distort the atrial anatomy and disrupt the conduction pathways.

### 4.3 PD in long‐term follow‐up

One‐third of the patients (*n* = 17, 34.7%), were non‐PD in a quindecinnial follow‐up. We were unable to identify any prior research focusing on the prevalence of PD in a long‐term follow‐up after OHT. An assessment of the atrial and ventricular pacing percentages (PP) revealed a relatively higher need for atrial electrical support in SND in comparison to the ventricular pacing percentages in AVCD (46.9 ± 40.6 vs. 24.0 ± 34.8, respectively), justifying the need for pacing in early and proximally located CD as previously reported.(Rivinius et al., 2019) The higher need for atrial support can be explained not only by significant bradycardia due to SND but also by the chronotropic incompetence of allografts.(Melton et al., 1999)

### 4.4 Prognostic assessment

Antecedent research regarding the prognosis in PPM was contradictory.(Cantillon et al., 2010; Rivinius et al., 2019) In comparison, our analysis of a very long‐term follow‐up after OHT showed PPM to be associated with poorer outcomes in univariate and multivariate logistic regression analyses. Nevertheless, as 90.0% of the SND and 87.9% of all CD requiring PPM were observed in biatrial OHT, the inferior prognosis might also be related to biatrial approach, although no explicit prognostic relevance was observed. Interestingly, after stratification according to the leading causes of death, we observed a significant correlation between PPM and septicemia but not with cardiovascular endpoints. Although we have no evidence for a direct association between the bloodstream infection and the device leads, implants in immunosuppression are, as known, associated with a higher mortality risk.(Paterson et al., 1998; Sherman‐Weber et al., 2004)

### 4.5 Observation period and PPM incidence

The recent guidelines on cardiac pacing and cardiac resynchronization therapy of the European Society of Cardiology advocate an observational period of at least 6 weeks for CD and chronotropic incompetence after heart transplantation.(Glikson et al., 2022) In contrast, according to the guidelines on cardiac pacing published in 2013, a period of clinical observation from 5 days up to some weeks was recommended.(European Society of Cardiology (ESC); European Heart Rhythm Association (EHRA) & Brignole, 2013) The extended observational period in the recent era may result in a lower rate of PPM requirement and in a preselection of the cases with persistent conduction disturbances in the immediate posttransplant period and beyond. Since we report on long‐term outcomes, most of our patients were transplanted in the first decade of the twenty first century. Thus, we gain a precious insight into the consequences of the recommendations in the earlier era. Additionally, we recognize how much progress has been made in this field in the last years.

## 5 LIMITATION AND STRENGTHS OF THE STUDY

The main limitation of our study is the small number of patients who had undergone device implantation. Additionally, there were insufficient data regarding the pretransplant factors, which might have a relevant influence on the posttransplant care and outcome. Nevertheless, when justifying the need for pacing in OHT and its consequences in follow‐up, we provide evidence based on echocardiographic and device assessment. Additionally, the evaluation of all factors was performed in an incomparable long‐term follow‐up after OHT.

## 6 CONCLUSIONS

One‐third of the OHT recipients with PPM are non‐PD in a long‐term follow‐up. However, PPM requirement is associated with poorer outcomes and correlates with fatal infectious complications. Notably, among the indications for PPM, the early SNDs are not only the most prominent but also the most persistent CD. Nevertheless, PPM implantation remains a case‐by‐case consideration because the donor and recipient determinants deliver no additional aid in identifying the subjects who might not require pacing in follow‐up. The surgical scar connecting the donor and recipient atria endure as the only factor, with unquestionable potential in building a bridge between the intrinsic conduction pathway and the device leads.

## CONFLICT OF INTEREST

The authors have no financial interests related to this manuscript.

## AUTHOR CONTRIBUTIONS

EA conception and design of the work, interpretation of the data, drafting the initial manuscript. CP, ADA and GF critical revision of the draft, IT, HR and JS supervision, interpretation of the data, critical revision for important intellectual content. All authors read and approved the version to be published.

## ETHICAL APPROVAL

The study was performed in compliance with the Declaration of Helsinki, and data sampling was approved by the local ethics committee (2019‐021‐f‐S).

## Data Availability

The data that support the findings of this study are available on request from the corresponding author. The data are not publicly available due to privacy or ethical restrictions.
